# Chronic Pain in Children: A Look at the Referral Process to a Pediatric Pain Clinic

**DOI:** 10.1155/2017/8769402

**Published:** 2017-03-22

**Authors:** Giovanni Cucchiaro, Jennifer Schwartz, Alec Hutchason, Beatriz Ornelas

**Affiliations:** ^1^Department of Anesthesiology and Critical Care Medicine, Children's Hospital Los Angeles, Los Angeles, CA, USA; ^2^Mental Health Center of Denver, Denver, CO, USA

## Abstract

We reviewed the referral pattern of children with chronic pain to a specialized pediatric pain clinic. Data were obtained from referring physicians and medical records and during an interview with patients and their parents by physicians and a psychologist. We analyzed the following: referral diagnosis, demographics, duration of symptoms, number of physicians previously consulted, school attendance, sports activities, presence of psychological disorders, final team diagnosis, and outcomes. Children had been experiencing pain for 34 ± 55 months. Patients had consulted on average 3 physicians in addition to their pediatrician. 32% of the patients had missed at least 10 days of school in a calendar year, and 47% had stopped playing sports. 15% had an operation because of pain that had been unsuccessful. The most common missed diagnosis was anxiety (25%) and depression (13%). 69% of the patients were back to school and/or playing sports within 4 months from our initial consultation. 32% of the patients did not make any progress during the follow-up period. The most common reasons for failure to improve were no compliance with the recommended treatments and poorly controlled major mood disorder. The time to refer children with chronic pain for specialized care could be extremely long causing significant social and psychological consequence.

## 1. Introduction

Literature data show that children with chronic pain often encounter significant delays between their initial pain complaints and the time they eventually consult a pain specialist [1–3]. Zernikow et al. have shown that children consulted on average 3 physicians prior to being referred to a pain specialist with most children having visited a doctor 1 to 5 times and about 13% of them having had a very high number of previous treatments and consultations (≥6) [3]. Konijnenberg et al. have reported a 12-month median duration of pain related symptoms and a median of 2 different physicians' consultations before children were referred to an academic pediatric specialist [4]. Reviews of trends in medicalization of children with chronic pain in the United States showed a longer duration of symptoms (18–24 months) and a higher level of medicalization, including a higher number of professionals seen (>8) compared to European studies [5, 6].

The reasons for these findings are still unclear. The current literature does not address how often an accurate diagnosis is made by general practitioners and nonpain specialist and whether the psychological and social components of pain are taken in consideration in the care plan. It is also unclear how the evaluation by a pain specialist can affect patients' outcomes, particularly in the presence of data showing that adherence to pain physicians' recommendations is inconsistent in part due to a negative attitude towards specific recommendations, in particular psychological interventions [7, 8].

The aim of this retrospective review was twofold. We analyzed the referral history for pediatric patients seen at our pain clinic, looking at the time interval between initial pain complaints and pain clinic consultation, including the number of physicians who consulted these patients and the referral diagnosis. We then analyzed the results of a multimodal approach to manage patients' symptoms and examined patients' compliance with the recommended treatments.

## 2. Methods

The study was reviewed and approved by the Institutional Review Board. We reviewed the medical record of patients seen in the pain clinic of an academic institution between October 2014 and June 2016. The inclusion criteria were children aged 3–20 years diagnosed with chronic headaches, neuropathic pain, and muscle-skeletal and chronic abdominal pain and patients whose prior medical record and note from the referring physicians were available. We excluded patients with cancer pain or acute onset pain defined as any pain lasting less than 12 weeks [9] and whose prior medical records were not available.

The pain clinic was staffed by a physician (anesthesiologist), a psychiatrist, a nurse practitioner, and a psychologist. A physical therapist and an occupational therapist were available for consultation as needed.

The duration of the initial encounter ranged between 90 and 120 minutes. During this time, Complete past medical, surgical, and family history were taken. This was followed by a psychological evaluation and a physical examination. Patients with neuromuscular deficits were also evaluated by physical therapists. The following data were extracted from the patients' electronic medical record: sociodemographic data, race and ethnicity, diagnoses, history of health care contacts including a list of consulting physicians, therapeutic procedures including surgeries, and ability of patient to practice sports and attend classes at school on regular basis.

We defined patients as unable to attend regular school classes when they were homeschooled because of pain symptoms and enrolled in online school or home-hospital programs and those who had missed more than 10% of school days during the previous school year, based on the California Department of Education policy [10].

Patients were asked about the time interval between the onset of pain symptoms and the referral to this pediatric pain clinic. A review of the patients' prior medical records and referral documentation confirmed the data's accuracy. We documented the type and number of providers consulted because of pain and we recorded whether patients were referred by a practitioner working at this institution or a physician practicing in the community.

Every patient was evaluated by a psychologist following a clinical interview assessing mental disorder classes and subcategories according to the Diagnostic and Statistical Manual of Mental Disorders, 4th Edition, Text Revision (DSM-IV-TR) [11] including depression, anxiety, behavior disorders, developmental disabilities, somatoform, substance use, and eating disorders. These interviews were conducted at the time of the patients' initial evaluation as well as during follow-up visits.

The recommendations made to patients and families included medications, psychotherapy, and Complementary and Alternative Medicine (CAM) interventions including acupuncture, yoga, biofeedback, massage therapy, and occupational therapy interventions. Nerve blocks and local or joint steroid infiltrations were also recommended when clinically indicated. Patients with Complex Regional Pain Syndrome and those unable to ambulate for chronic pain were admitted in the hospital rehabilitation unit for intense physical and occupational therapy. Patients with history of opioid abuse were referred to the hospital substance abuse program specialists.

Patients were asked to follow up with the clinic within a month after the initiation of the recommended treatments. The following appointments were scheduled based on the response to the treatments. Phone calls were made to remind patients of their clinical appointments. Treatments including psychotherapy and physical and occupational therapy as well as CAM interventions were done at this institution or in the community based on patients' insurance requirements and their physical address.

We defined clinical improvement in our population as the ability of patients to go back to school full time as well as being able to resume sport activities that were interrupted because of pain [12, 13].

### 2.1. Statistical Analysis

STATA/IC 14 software was used for data analysis (StataCorp LP, College Station, TX, USA). Chi-square analysis was conducted to compare categorical data after verification of their normal distribution using the Silk-Wilk test. A multivariable least square regression model was used to analyze the independent relationship between duration of symptoms and potential different predictors of delayed referral to our pain clinic including distance from our hospital, type of insurance, referral diagnosis, and race. A similar analysis was conducted to determine whether any of variables could predict the number of doctors' visits done prior to our evaluation. *p* values < 0.05 were considered statistically significant.

## 3. Results

### 3.1. Population Characteristics

Seventy-five patients were included in the review. Demographic data are shown in Table 1. A greater number of female than male patients were referred to the pain clinic (*p* < 0.01), during the study period, there were fewer Hispanic patients compared to non-Hispanic patients (*p* < 0.001), and only 5% of the families did not speak English and needed a translator during the visit (*p* < 0.001). Thirty-nine children (52%) were not able to attend regular classes because of pain and 35 (47%) were unable to continue playing sports because of pain (Table 1).

Patients had been reporting pain for an average of 34 ± 55 months. The duration of symptoms was twice as long in patients who had a Health Maintenance Organization (HMO) insurance plan and Medi-Cal compared to patients who had a Preferred Provider Organization (PPO) or Kaiser insurance plan (42 ± 44 versus 21 ± 18 months, resp., *p* < 0.05). The longest duration of symptoms prior to the referral to our pain clinic was observed in patients with headaches (61 ± 58 months), followed by abdominal pain (39 ± 33 months) and back pain (37 ± 55 months). Patients diagnosed with dystonia and fibromyalgia had the shortest referral time (8 ± 5 months).

The referral diagnoses are listed in Table 2. The most common reason for referral was chronic muscle-skeletal pain, followed by abdominal pain, back pain, and headaches. The list of specialties of the referring physicians is shown in Table 3. There was no statistical difference in delay to refer to this pain clinic among the different referring physicians (range 25 to 41 months).

Patients consulted an average of 3 specialists in addition to their pediatrician before being referred to the pain clinic (range 1–8 specialists), and only 4 patients never saw their pediatrician (5%). The most commonly consulted specialists were as follows: orthopedic surgeons (41%), rheumatologists (27%), and neurologists and gastroenterologists (24%). Ten percent of the patients had at least one emergency room visit since the pain symptoms had started with 2 visits (range 1–4) on average prior to the referral to our clinic. There was no correlation between the number of specialists consulted and the delay to refer patients to this clinic. The multivariate regression analysis identified distance to our clinic and type of health insurance as the variables that predicted a delay in referring patients to our pain clinic (Table 4).

Sixteen patients (21%) had consulted a psychologist prior to visiting our pain clinic. Four patients were seen for anxiety, 5 for depressive symptoms, and 6 either after parents divorced or after episodes of trauma (i.e., bullying, physical assault). In only one of these patients psychotherapy had been recommended to help manage chronic pain symptoms.

Eleven patients (15%) underwent an operation because of pain complaints prior to the referral to the pain clinic. The list of procedures includes 2 appendectomies, 1 Nissen fundoplication and 1 cholecystectomy done for complaints of chronic abdominal pain, 2 foot surgeries for pain thought to be related to feet deformities, and 1 resection of a small leg osteochondroma for chronic leg pain. All of these patients continued to report pain after the aforementioned operations similar in intensity and quality to that experienced prior to their operation.

### 3.2. Outcomes

The types of interventions recommended by our team are shown in Table 5. We confirmed the referral diagnosis in 22 patients (29%). In 34 patients (45%) we identified psychological factors as a major contributor to pain and disability: 19 patients were diagnosed with anxiety, 13 were diagnosed with depression, 1 was diagnosed with psychosis, and 1 was diagnosed with somatization disorder. We confirmed the diagnosis of CRPS in 4 of the 6 patients referred for CRPS of the lower extremity. These patients were admitted in our chronic pain inpatient rehabilitation program, while the remaining 2 patients were diagnosed with generalized anxiety disorder and referred for psychotherapy.

We prescribed SSRI antidepressants to 7 patients. Six of them improved during the follow-up period. The patient who failed the recommended treatment also had a diagnosis of opioid abuse. We recommended psychotherapy in conjunction with CAM therapies in 24 patients. Three of them did not come back to the clinic, 14 followed the recommendations, and all of them improved during the follow-up period. Seven patients chose not to consult a psychologist; subsequently, none of these patients reported improvements in their pain symptoms.

Our team misdiagnosed a patient. He was initially diagnosed by surgeons with chronic functional abdominal pain. Because of negative physical findings we recommend CAM. However, after further investigations conducted by his surgeon, he was found to have an intra-abdominal neuroblastoma 6 months later.

The length of the follow-up was 11 ± 10 months. Twelve patients (16%) decided not to come back to this pain clinic and were lost to follow-up. The average number of follow-up visits was 3 (range 1–20). Of those who returned to the clinic for follow-up, 17 patients (27%) did not follow up with the recommended treatments. Of those who adhered to the treatment plan, 43 patients (68%) were functional and had returned to their daily activities either pain-free or able to cope with their symptoms. In particular, there were a significant higher number of children who were able to go back to regular classes during the follow-up period compared to the time prior to our evaluation and recommendations (85% versus 50%; *p* < 0.0001). Similarly, 62% of our patients were practicing a sport activity after having followed our treatment plan compared to 20% prior to it (*p* < 0.0001). It should be noticed that 36% of the children who were not interested in practicing any sport at the time of the initial clinic visit started practicing some sport as part of our overall recommendations.

The interval between the initial evaluation and clinical improvement was 4 ± 3.5 months. The pain symptoms in this group of patients had lasted on average 37 months prior to being seen in our clinic. Twenty patients (32%) reported no improvement in their symptoms. This lack of improvement has been attributed to one of the following: wrong diagnosis in 1 patient made by this team, drug abuse and dependence in 2 patients, lack of insurance for 2 patients, no compliance with the recommended treatment in 7 patients, and major depression or generalized anxiety poorly controlled by psychotherapy and medications in 7 patients.

## 4. Discussion

This review highlights two important aspects of the care delivered to children with chronic pain: the delay to refer these patients to a pediatric pain specialist and the failure to recognize psychological disorders as an important comorbid condition in chronic pain in children and adolescents.

We found that the delay to refer children with chronic pain to our clinic was significantly longer than what has been reported in other studies [5, 6]. The reason for this difference is unclear. Our data indicate that the type of insurance patients carry may be a contributing factor to a delayed first contact with a pain specialist. In particular, patients with Medi-Cal or HMO type of insurance coverage waited twice as long as compared to patients who had PPO plan. The referral process in the United States health care system can be quite complex and it is triggered in the majority of cases by a general practitioner or a pediatrician. The request for a specialist consultation is then reviewed by insurance companies that may approve or deny it. Although the time for preauthorization approval is similar for Medi-Cal as well as other insurance plans (10 versus 3–5 days, resp.) [14], insurance companies can deny a specific referral. We did not have any information on the insurance companies' review decision processes. A 2003 study on private insurance patients covered by two California medical groups showed that the denial rate for physician services ranged between 11 and 24% [15].

We have confirmed literature data [16] suggesting that distance to specialized health care can be a barrier to access resulting in a late and inaccurate diagnosis and management of children with chronic pain.

Lack of specialized care and low household income are other major barriers to access specialized care in general [17, 18]. The presence of limited resources available in communities to manage children with chronic pain has been shown to be a significant barrier in several countries [19–21]. There are only two pediatric pain comprehensive programs in the Los Angeles County (population 2,325,047 according to the 2015 census) [22] which can explain the delay to access our services: the average waiting time for an evaluation at our clinic is 7 weeks. Our data seems also to support reports showing that low-income household may face additional barriers in accessing pain specialists. We were not able to assess our families' income and this may limit our conclusions. However, the type of health care insurance is often a reliable indirect indicator of household income in the United States, with families in the lower income brackets usually insured by governmental or HMO plans, which have often limited options with respect to specialized health care providers. The majority of our patients (72%) were enrolled in such plans (Table 1).

Although the type of insurance and distance were significantly correlated to the delayed referral to the clinic, the model accounted only for 10% of the findings. When looking at other potential reasons for the delayed referral it is important to consider the pediatricians', specialists', and families' biases towards the search for an organic cause of pain in children. It is difficult to quantify how much inappropriate referral practices can be attributed to the belief that there are definitive tests for every specific disease and that causal determinism can be explained with certitude in medicine [23]. It is quite concerning that the quest for more diagnostic tests and specialists could lead to operations as shown in our series (15% of the patients) which did not resolve the patients pain complaints. Moreover, our data confirm previous reports that support waiting as little as five weeks before establishing an appropriate set of interventions to manage chronic pain results in declining health related quality of life [24].

With respect to health care utilization, our data are consistent with other reports. While a study found that higher income, more severe pain scores, and activity limitations were significant predictors of the number of doctors' visits [25], we could not identify a significant predictor among the variables we evaluated.

Even though the biopsychosocial model of chronic pain is widely accepted by chronic pain practitioners, it is often difficult for families to accept that psychological and social factors intricately interrelate to affect pain perception, coping with symptoms, engagement with providers, and, ultimately, clinical outcomes. We identified anxiety and/or depression in 45% of our patients. Longitudinal studies have clearly indicated that depression and anxiety are risk factors for developing chronic pain [26, 27]. At the same time chronic pain can trigger depression, fear, and anxiety with significant behavioral consequences, functional limitation, and perceived disability [28, 29]. The high prevalence of psychological disorders in our patients, similar to that reported in other studies [30, 31], confirms the importance of integrating a psychologist in the team of practitioners treating children with chronic pain. Some of our patients were already receiving psychological support from a psychologist before being referred to our clinic. However, this was an isolate intervention without the support of multiple integrated services further emphasizing the importance of a multidisciplinary approach to chronic pain.

Not only is it important to recognize the appropriate time to refer a child with chronic pain to a pain specialist but also it is important to identify what kind of pain practice the child should be referred to. There are different types of pain clinics, those where symptom control is the priority and others that focus on improving patients' functional activities. It is rare that both approaches are used [32].

Adherence with physicians' recommendations is often a major obstacle to clinical improvement. The number of patients who did not come back to the clinic or did not adhere to the treatment plan (36%) was slightly lower than what has been reported in other studies focused on chronic pain (47%) [33]. Surprisingly, Hechler et al. reported a significant improvement in patients who did not attend their clinic as recommended, opposite to our findings [33]. We failed to see any improvement in 32% of our patients. This lack of improvement could be attributed to relatively short follow-ups (11 months), the high percentage of patients with severe psychiatric disorders and drug abuse (12%), conditions that may have require prolonged interventions, and the limited compliance with the recommended treatments. Patients who complied with our recommended treatments returned to school and were able to socialize again with their peers within a short period of time (4 months) in relation to the length of their symptoms. These data are in line with those from other reports where a multidisciplinary approach by pain specialists has been utilized to address chronic pain in adolescents [34, 35].

There are multiple reasons why we observed a poor compliance as well as a relatively high number of patients who did not follow up with the recommended treatments. Patients may have faced problems obtaining insurance coverage for some of the CAM interventions we recommended. Opposite to data obtained in the adult population where a recent study has shown that 30–40% of adults in the United States use some form of CAM intervention every year [36], a significantly lower number of children and adolescents (12%) utilize CAM interventions [37]. The reasons for this different behavior could again be explained by parents' understanding and attitude towards chronic pain in children and their search for a medically based solution. Adolescents' limited acceptance of psychotherapy and poor adherence to treatment protocols are also a well-documented phenomenon that may have contributed to our findings [38].

This study has several limitations. This was a retrospective analysis and we could not report on the recommended outcomes proposed by the PedIMPACT [39] focus group because of missing data. It should be recognized that lack of time, lack of money, and lack of manpower are obvious barriers to the routine implementation of the PedIMPACT outcomes measurements in clinical practice [40]. We were though able to report on two criteria often utilized as indexes of functional improvement (return to school and sport activities) [12] in every patient we followed up. Although the type of insurance patients carried seemed to be a barrier to access our pain clinic we were not able to determine whether the denial of service was the most important determinant for the delayed referral. Some of our patients were already receiving psychological support from a psychologist before being referred to our clinic. Our data do not allow us to explain why previous psychological interventions were ineffective. We can only speculate whether this was due to the fact that the psychological support was an isolate intervention without the support of multiple integrated services. Although we reported the level of adherence to the recommended treatments, we do not have enough data to identify the exact cause of this behavior. Also, this review does not answer the question about the exact timing for the referral to a pain clinic. It seems that physicians should take into consideration a specialized evaluation and a multidisciplinary approach in the presence of stalled progress and persistent disability.

In conclusion, our data support the notion that there is a significant delay in referring children with chronic pain to pediatric pain specialists. The psychological component of pain is often missed, and this results in multiple doctor and emergency room visits, multiple laboratory and radiologic tests, and unnecessary operations with consequences on the quality of life of children. A multidisciplinary evaluation and a broad range of interventions including CAM techniques, psychotherapy, and referral to appropriate pediatric specialists could significantly expedite the return to a functional life with well managed pain symptoms. Although some of the academic pediatric pain clinics housed in large medical centers utilize a multidisciplinary or interdisciplinary approach, our data support the need for providing mental and behavioral health services as part of a primary care model as psychologists play a key role in integrated health care by helping people modify their behavior to prevent and recover from health problems.

## Figures and Tables

**Table 1:** Demographic data of the study population (CHLA: Children's Hospital Los Angeles; HMO: Health Maintenance Organization).

	*N*	%	*p*
*Speaking English*			
Yes	71	95%	0.001
No	4	5%	
*Age*	13 ± 3 range (6–19)		
*Sex *			
M	28	29%	0.002
F	47	71%	
*Ethnicity: *			
Hispanic	22	29%	0.001
Caucasians	53	71%	
*Duration of symptoms (months)*	34 ± 37 (range 3–199)		
*School attendance:*			
Missing classes-homeschooled	39	32%	
*Practicing sports: *			
No for pain	35	47%	
*Distance from CHLA (miles)*	33 ± 39		
*Referral *			
CHLA	34	45%	0.253
Community physicians	41	55%	
*Insurance *			
Private	21	28%	0.186
Government	31	41%	
HMO	23	31%	
*# of specialists consulted prior to CHLA visit*	3 ± 2 range (1–8)		

**Table 2:** List of referral diagnoses (CRPS: Chronic Regional Pain Syndrome).

Diagnosis	*N*	Percentage
Musculoskeletal pain	27	36%
Abdominal pain	15	20%
Back pain	12	16%
Headache	8	11%
CRPS	5	7%
Chest pain	3	4%
Dystonia	2	3%
Sickle cell disease	2	3%

**Table 3:** List of referring physicians by specialties.

Referring physicians	*N*	%
Pediatrician	34	46%
Orthopedic surgery	13	17%
Rheumatology	9	12%
Gastroenterology	7	9%
Neurology	6	8%
Surgery	2	3%
Hematology	2	3%
Endocrinology	1	1%
Pulmonologist	1	1%

**Table 4:** Multivariable least square regression analysis exploring the association between duration of symptoms and type of insurance, distance from our hospital, referral diagnosis, and race.

	Coefficient	Std error	*t*-value	95% CI		*p* value
Distance	0.31	0.14	2.25	.0351	.589	0.028
Type of insurance	−0.55	0.25	−2.2	−1.043	−.049	0.032
Diagnosis	0.02	0.04	0.43	−.066	.102	0.67
Race	0.04	0.26	−0.17	−.482	.570	0.67

**Table 5:** Therapeutic interventions recommended by our team with outcomes (CAM: Complementary Alternative Medicine).

New recommendations	*N*	Improvement	Lost to follow-up
CAM	14	8 (57%)	3 (21%)
CAM + psychotherapy	25	14 (56%)	3 (8%)
Admission in Chronic Pain Rehabilitation program	7	5 (71%)	0
Referral to surgical services	7	4 (57%)	1 (14%)
Referral to medical services	3		3 (100%)
Spinal cord stimulator, pump for intrathecal delivery of morphine	2	2 (100%)	0
Local or epidural infiltration with steroids	4	2 (50%)	1 (25%)
Referral to physical therapy	3	1 (33%)	0
Antidepressant	7	6 (86%)	0
New opioid-anticonvulsant	3	1 (33%)	1 (33%)
*Total*	*75*	*43 (57%)*	*12 (16%)*

## Abbreviations

CAM: Complementary and Alternative Medicine.
